# Changes in Distance between a Wearable Robotic Exoskeleton User and Four-Wheeled Walker during Gait in Level and Slope Conditions: Implications for Fall Prevention Systems

**DOI:** 10.3390/biomimetics8020213

**Published:** 2023-05-23

**Authors:** Koki Tan, Soichiro Koyama, Hiroaki Sakurai, Yoshikiyo Kanada, Shigeo Tanabe

**Affiliations:** 1 Graduate School of Health Sciences, Fujita Health University, Toyoake 470-1192, Aichi, Japan; koki.tan@fujita-hu.ac.jp (K.T.); hsakurai@fujita-hu.ac.jp (H.S.); yokanada@fujita-hu.ac.jp (Y.K.); tanabes@fujita-hu.ac.jp (S.T.); 2 Faculty of Rehabilitation, School of Health Sciences, Fujita Health University, Toyoake 470-1192, Aichi, Japan

**Keywords:** rehabilitation robotics, assistive device, fall prevention, gait training, powered exoskeleton, slope

## Abstract

When walking with wearable robotic exoskeletons (WRE) in people with spinal cord injury, the distance between the user and the walker is one of the most important perspectives for ensuring safety. The purpose of this study was to clarify the distance between WRE users and four-wheeled walkers (4WW) while walking on level and sloping surfaces. To eliminate the effects of variation in neurological conditions, 12 healthy subjects participated. All participants ambulated using the WRE and the 4WW on level and sloping surfaces. The outcomes were the mean distances between the WRE users and the 4WWs in the level and slope conditions. To examine the influence of uphill and downhill slopes on distance, comparisons were conducted between the uphill or downhill conditions and the respective transitional periods. In the uphill condition, the mean distances were significantly greater than that in the level condition. Conversely, the mean distance moving downhill was significantly shorter than that in the level condition. Changes in the distance between the WRE user and the 4WW might increase the risk of falling forward on an uphill slope and backward on a downhill slope. This study’s results will assist in developing a new feedback system to prevent falls.

## 1. Introduction

Spinal cord injury (SCI) is caused by a traumatic event, with the incidence of new cases at 934,951 (780,963 to 1,155,187), and age-standardized incidence rates of 13 (11 to 16) per 100,000 worldwide in 2016 [1]. Despite rehabilitation, several patients remain unable to walk without support [2]. An opportunity for standing and walking reduces medical problems (e.g., pressure sores, osteoporosis, spasticity, and edema) caused by long-term sitting on a wheelchair [3,4,5].

Wearable robotic exoskeletons (WRE) are powered exoskeletal devices that enable walking in patients with SCI [6,7,8,9]. WREs are capable of generating multiple step repetitions while bearing full weight on the paralyzed lower limbs. Some patients, as reported in previous clinical trials, were able to walk continuously for more than 60 min and approximately 1500 m using WREs without assistance [10,11].

Some clinical trials on walking with WREs are being conducted, not only on level ground, but also in a variety of indoor and outdoor situations in patients with SCI. A previous study reported that patients with SCI, specifically, paraplegia (T4-L1), were able to walk outdoors with a forearm crutch and ReWalk, under supervision [12]. Hartigan et al. [13] showed that patients with tetraplegia (C5-7) were able to walk on various indoor and outdoor surfaces, including elevators, sidewalks, thresholds, carpets, hard flooring, and slopes compliant with the Americans with Disabilities Act, with assistance of one or more people. Another study determined that five patients with SCI (C4-T10) were able to walk on the carpet; another five patients walked up and down slopes with grades of 5%–8% with minimal-to-moderate assistance [10].

Some patients with SCI who use WREs during walking use a four-wheeled walker (4WW) to aid with mobility [14]. A 4WW is commonly used to maintain postural stability in patients with muscular weakness in the lower limbs and balance impairments [15]. Regarding the configuration of the walking aids, a greater support base is required for patients with poor postural stability [16]. Although a 4WW has a greater support base and higher stability, it is necessary for the WRE user to maintain the proper distance between themselves and the 4WW.

A previous study in patients with SCI reported that unexpected postural changes occurred in the early stages of walking practice using a WRE with a 4WW on level ground because of the changing distance between the WRE user and the 4WW [17]. The study reported that the risk of falls was increased by excessively long and short distances between the WRE user and the 4WW. In particular, a long distance caused a forward postural change, and a short distance caused a backward postural change in patients with SCI [17]. If the distance between the WRE user and the 4WW is too great, the resulting hyperextension of the user’s hip joints may lead to a forward fall. In contrast, if the distance between the WRE user and the 4WW is too short, the user’s hip joints may become hyperflexed, leading to a backward fall.

Previous studies have suggested the specificities of walking on slopes. The risk of falling on a slope is higher than that on level ground or stairs [18]. Patients with fractures of the femur have difficulty operating 4WWs and controlling their acceleration during slope walking [19]. Biomechanical research of slope walking is important to understand the causes of falls in outdoor walking [20]; however, changes in walker position when using WREs in level and slope conditions and transitions between them have not been fully clarified. When walking uphill, the force of gravity shifts the 4WW backward, requiring the user to apply more force to push the walker uphill. When walking downhill, gravity shifts the 4WW forward, which means that the user will need to hold the walker back to keep from moving too quickly. When walking on slopes, these changing forces result in variable distances between the WRE user and the 4WW.

The purpose of the present study was to clarify the changes in distance between the WRE user and the 4WW while walking uphill or downhill in the level and slope conditions and while transitioning between level and slope conditions. The present study was conducted with healthy persons to eliminate the effects of neurological level and motor or sensory function (e.g., ASIA and Frankel Classification). Clarifying this point would help understand the risk of falling while walking on a slope and contribute to the development of a fall prevention feedback system to facilitate walking practice with WREs.

## 2. Materials and Methods

### 2.1. Study Design and Participants

This study had a cross-sectional design. In total, 12 healthy volunteers with a mean age of 23.8 years (standard deviation [SD] 4.9; 2 women) participated in this study, using convenience sampling. The study was conducted in accordance with the Declaration of Helsinki, and the study protocol was approved by the Institutional Ethics Committee (approval no. HM19-283). Before initiating the experiment, the participants were provided with an explanation of the study objectives and procedures to receive their consent. Written informed consent was received from all participants.

### 2.2. Experimental Setup

**Figure 1:**
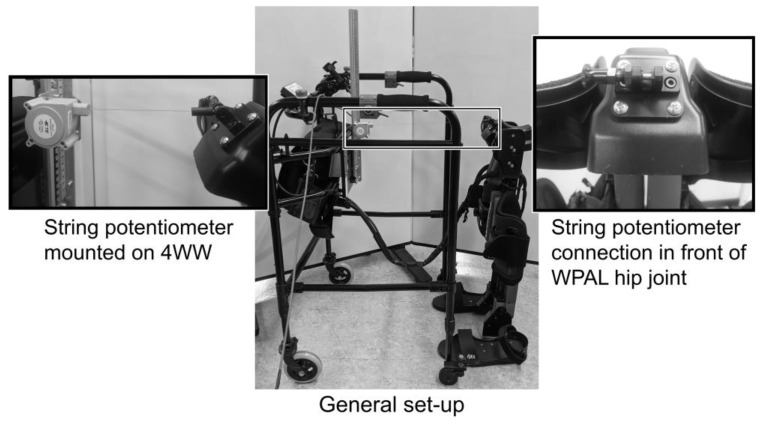
The setup for measuring distance between the four-wheeled walker (4WW) and the wearable robotic exoskeleton (WRE) user. SP2-50 TE Connectivity (Schaffhausen, Switzerland) was used as the string potentiometer. The string potentiometer was mounted on the front of the 4WW at the same height as that of the WPAL hip joint.

For the walking track, a level ground and slope were set up in a laboratory. The level ground was 10 m long. The slope was inclined at 10% and was 4.25 m long and 850 mm wide. The experimental setup consisted of a Wearable Power-Assist Locomotor (WPAL [WPAL-G; ASKA Corp., Aichi, Japan]), a specialized 4WW for WPAL, and a string potentiometer (SP2-50; TE Connectivity, Schaffhausen, Switzerland). Figure 1 shows the general setup for measuring the distance between the WRE user and the 4WW. The distance between the WRE user and the 4WW during gait was measured using the string potentiometer. The TE Connectivity SP2-50 string potentiometer consisted of a rugged polycarbonate enclosure and measured linear distances up to 1270 mm. The string potentiometer was mounted on the front of the 4WW at the same height as that of the WPAL hip joint.

The height of the walker was adjusted to the height of the hand with 0° of shoulder flexion and 20° of elbow flexion in the upright position, as determined in a previous study [21]. A personal computer with an A/D converter (USB-6343, National Instruments, Austin, TX, USA) and the LabVIEW 2019 software (National Instruments, Austin, TX, USA) was used to record data from the string potentiometer, with a sampling frequency of 1 kHz.

### 2.3. WPAL and Specialized 4WW

The WPAL is a WRE designed for walking for patients with SCI [7,11,22,23,24,25]. The WPAL was used with a specialized 4WW. A thin wire cable linked the 4WW to the WPAL. The details of the WPAL and the specialized 4WW are described below.

The main components of the WPAL include six motors and frames to mount them, located between the lower limbs. A mechanical hip joint is attached to the frame under the perineum and is connected medially to a sliding structure, to align the virtual center of rotation of the robotic hip joint with the physiological center of the hip joint. Independently controlled motors mounted on the hip, knee, and ankle joints provide specific ranges of motion (hip, 40° [flexion 25°–extension 15º]; knee, 120° [flexion 120°–extension 0°]; and ankle, 50° [dorsiflexion 35°–plantar flexion 15°]). Custom-made brushless DC servo motors are used in each joint, with the overall device weighing approximately 13 kg. The WPAL can be used by persons with a weight of up to 80 kg and a height of 145–180 cm.

Regarding the 4WW specifications, weight of the 4WW is approximately 12 kg, width is 540 mm, and height ranges from 850 to 1000 mm. The wearable unit of the WPAL has a central processing unit, motor drivers, and batteries responsible for controlling the actuators. This unit can be placed on the 4WW. Placing the motor control circuitry and batteries in the 4WW ensures safety and eliminates the need for users to carry the device themselves. The rear wheels of the 4WW have a ratchet mechanism to prevent backward rotation. The 4WW has handgrips on both sides equipped with button and lever switches for users to operate independently. Skilled users can wear and remove the device in under 2 min.

### 2.4. WPAL Operation Using a Specialized 4WW

The operation details of the WPAL have been published earlier [26]. Briefly, the WPAL has five modes, herein listed in the order of their use: manipulating the robot’s knee joint angle, standing up, manipulating robot’s ankle joint angle, walking, and sitting down. The user selects each mode by pressing a button on the left grip of the specialized 4WW. Before the walking trials, the length of both lower limb parts of the WPAL is adjusted to match the length of the user’s lower limbs. To wear the robot, the user, in a wheelchair, approaches the robot waiting in a standing position. Next, the user places both feet on the foot plates of the robot and fastens them with bands. For manipulating the robot’s knee joint angle to wear the robot, the user pulls the lever attached under the grip of the 4WW. This operation makes the robot’s knee joint bend from the fully extended position to the knee joint angle of the user in a wheelchair sitting position. When the robot frame and the axis of the user’s lower limbs are parallel, the user fastens them with bands. In the standing up mode, the user stands up by pressing a button on the right grip and shifting their center of gravity slightly forward using voluntary movements of the upper limbs and trunk. Immediately after the forward leaning motion, the robot’s knee joints move to the fully extended position and the robot’s ankle joints move to the intermediate position. As a result, the user is in a standing position. To manipulate the robot’s ankle joint angle mode, by pulling a lever, the user can adjust the angle of the robot’s ankle joint to maintain a steady posture while standing. Prior to the start of walking, some parameters must be set. These parameters include double support time (0.1 to 1.0 s), swing time (0.9 to 1.5 s), toe clearance height (0 to 20 mm), and stride length (0 to 700 mm). The gait speed is calculated as the product of stride length and swing time, with a maximum achievable speed of 1.3 km/h. For safety reasons, the user does not operate the computer themself. Then, the WPAL moves the lower limbs in a continuous alternating pattern, while the user uses trunk and upper limb muscles to laterally shift their center of gravity in response to the motion of WPAL in the walking mode. To initiate the sitting down motion, users press the right button. Finally, to manipulate the robot’s knee joint angle to remove the robot, the user pulls the lever attached under the 4WW grip again.

WPAL has various gait patterns, including a “slope mode”. In the “slope mode”, the robot’s ankle joint angle changes automatically from normal walking mode after the detection of inclination measured by a tilt sensor installed on the 4WW. While walking on the slope, the robot’s ankle joint angle during the stance phase is adjusted according to the moving average of the inclination of the 4WW.

### 2.5. Experimental Procedure

The WRE and the 4WW were used simultaneously during the experiment. All participants practiced sufficiently by walking with the WPAL and the 4WW until they were able to walk independently on level and sloping surfaces. First, the participants walked on level ground. When they were able to walk safely and independently, measurements were also taken on slopes. In the measurements on the slope, the participants walked uphill first, followed by downhill. When participants stopped on the slope, the experiment assistant helped them down the slope to a safe place. Once they could walk independently, they were then asked to walk on a 10 m level surface and a 4.25 m slope (uphill and downhill), once each. The distance between the WRE user and the 4WW was measured while walking in each condition. During the experiment, the general setup was used to measure the temporal changes of distance between the WRE user and the 4WW on both level and sloping surfaces. The gait parameters of the WPAL in normal walking mode were as follows: swing time 0.8 s, double support time 0.4 s, and stride length 560 mm. The robot’s ankle joint angle on level ground was set to 0°.

### 2.6. Data and Statistical Analysis

**Figure 2:**
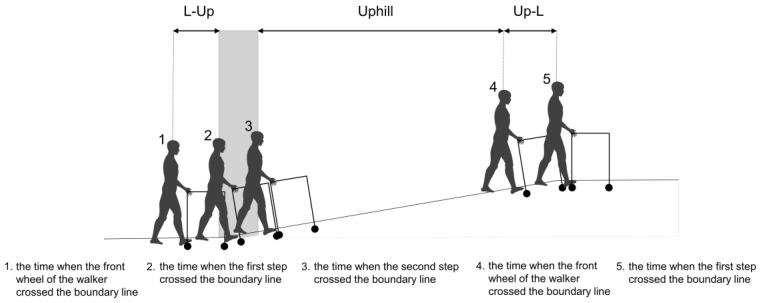
Definitions for classifying the slope walking path into six sections. The uphill and downhill conditions were defined as the periods from the time when the second step crossed the boundary line to the time when the front wheel of the four-wheeled walker crossed the boundary line. The transition between level and slope (L-Up, Up-L, L-Dn, and Dn-L) was defined as the period from the time when the front wheel of the four-wheeled walker crossed the boundary line to the time when the first step crossed the boundary line. The slope was inclined at 10%.

Seven conditions were retrieved from all distance data as follows: (1) level; (2) level to uphill (L-Up); (3) uphill; (4) uphill to level (Up-L); (5) level to downhill (L-Dn); (6) downhill; and (7) downhill to level (Dn-L) (Figure 2). For the level condition, six gait cycles, excluding the first three steps, were used for the analysis. The uphill and downhill conditions were defined as the periods from the time of the second step crossing the boundary line from level to slope to the time of the step immediately before crossing the front wheel of the walker over the boundary line from slope to level. The transition between level and slope included L-Up, Up-L, L-Dn, and Dn-L, which were defined as the period from the time of the step immediately before crossing the front wheel of the walker over the boundary line to the time of the first step crossing the boundary line. Each mean distance was calculated from continuous time-series distance data. A paired-samples t-test was conducted for comparison between uphill and downhill slopes. Repeated one-way analysis of variance (ANOVA) was used to analyze the influence of the uphill slope (level, L-Up, uphill, and Up-L) and downhill slope (level, L-Dn, downhill, and Dn-L) on the distance between the WRE user and the 4WW. If there was a significant main effect, a post hoc test was performed using multiple two-tailed t-tests with a Bonferroni correction for multiple comparisons. Statistical analyses were performed using the R software version 4.0.3. The statistical significance level was set at a *p*-value < 0.05.

## 3. Results

**Figure 3:**
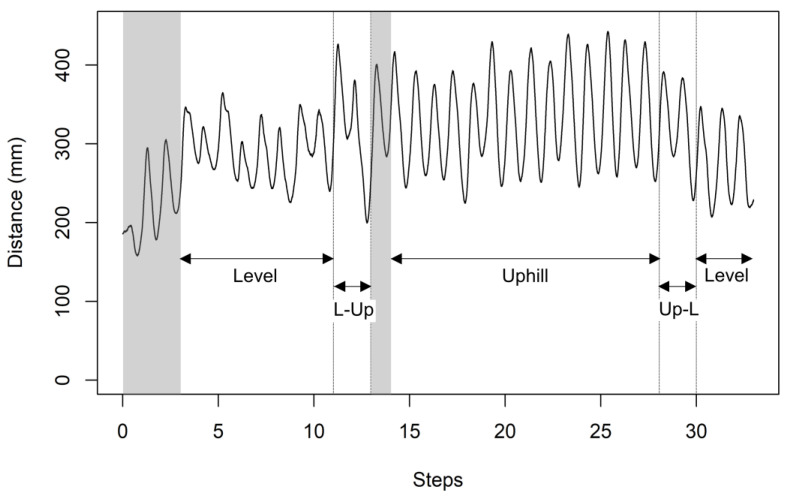
Raw data of distance from one participant walking in uphill condition. This data shows temporal changes in the distance between the WRE user and the 4WW. The dotted line shows the transition between level and slope conditions (L-Up and Up-L). L-Up, level to uphill; Up-L, uphill to level. Gray shading indicates excluded data (i.e., data during the first three steps of level surface and the first step of the slope).

**Figure 4:**
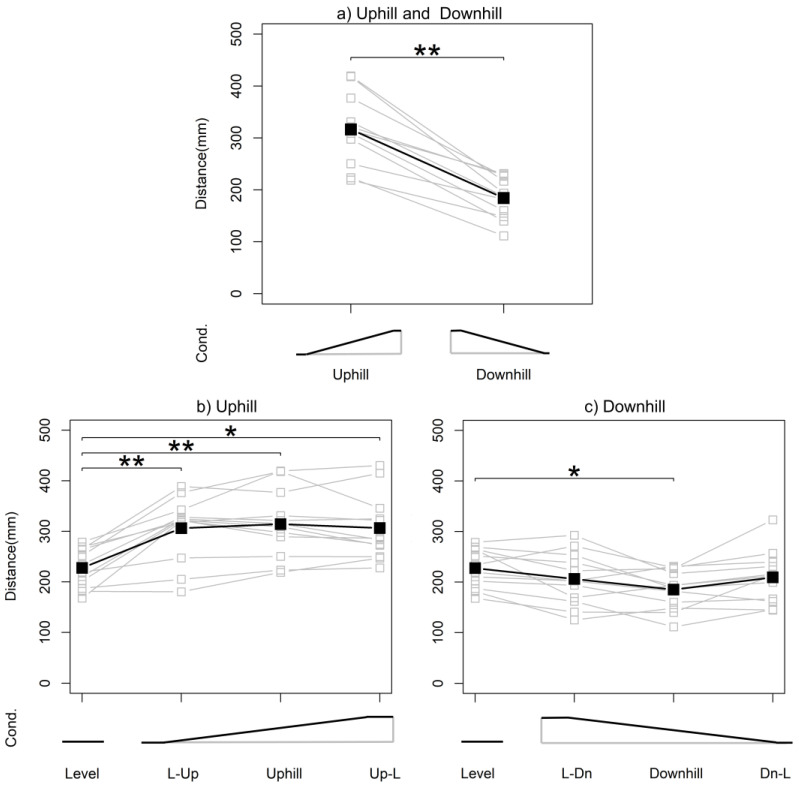
Mean distance for each condition (cond.). An asterisk indicates *p* < 0.005, and a double asterisk indicates *p* < 0.001. L-Up, level to uphill; Up-L, uphill to level; L-Dn, level to downhill; Dn-L, downhill to level; cond., condition.

All participants completed all experimental tasks without dropping out. There were no adverse events, including minor injuries, during the experiment. When walking with the WPAL, the subjects performed movements such as pushing a 4WW to move forward and stopping a 4WW to support their body, shortening and lengthening the distance between the WRE user and 4WW accordingly. Figure 3 shows a row of data of distances between the WRE user and the 4WW. The mean distances between the WRE user and the 4WW were 227.6 mm on level, 306.2 mm on L-Up, 314.2 mm on uphill, 306.5 mm on Up-L, 206.1 mm on L-Dn, 185.0 mm on downhill, and 209.2 mm on Dn-L. Figure 4 shows a comparison of the mean distances for each condition. Paired-samples t-tests revealed significant differences between the uphill and downhill slopes (t_(11)_ = 8.8246, *p* < 0.0001). Repeated ANOVA revealed significant differences between the uphill slope conditions (level, L-Up, uphill, and Up-L [F_(3,33)_ = 23.12, *p* < 0.0001]) and between the downhill slope conditions (level, L-Dn, downhill, and Dn-L [F_(3,33)_ = 4.8629, *p* = 0.00655]). In the post hoc test, mean distances in the uphill, L-Up, and Up-L conditions were significantly increased compared to the level condition (*p* = 0.00035 [uphill vs. level], *p* = 0.00114 [L-Up vs. level], *p* = 0.00027 [Up-L vs. level]). In contrast, the mean distance in the downhill condition was significantly decreased compared to the level condition (*p* = 0.0026).

## 4. Discussion

Our results showed that the distance between the WRE user and the 4WW varied during level and slope walking conditions, as well as in the transitional periods. Specifically, the distance between the WRE user and the 4WW increased during uphill conditions including transition between level and slope (uphill, L-Up, and Up-L) and decreased during downhill condition not including transition between level and slope.

These changes in the distance between the WRE user and the 4WW might result from the fact that the distance between the proximal upper limb (i.e., the shoulder joint) and the WRE handgrip remained relatively constant during the level walking condition, whereas the distance between the WRE user and the 4WW might have been influenced by two factors during the slope conditions. The first factor is the height of the 4WW handgrips. The handgrip height changes from a higher position on the uphill slope to a lower position on the downhill slope. A higher handgrip position implies that the proximal upper limb is closer to the handgrip, which might lead participants to position the 4WW at a greater distance from themselves to maintain a stable distance between the WRE user and 4WW. The second factor is the trunk position of the WRE user. Under the slope conditions, the force parallel to the slope was generated by the weight of the 4WW (approximately 12 kg). The inclination of the slope was 10%, resulting in an 11.6 N backward force on the uphill slope and an 11.6 N forward force on the downhill slope. As mentioned above, to maintain a stable distance between the WRE user and 4WW against these forces, the WRE users must lean their trunks forward on the uphill slope to easily push the 4WW and backward on the downhill slope to easily pull the 4WW. Consequently, because the length of the upper limb is constant, the WRE users might operate the 4WW in a distant position on the uphill slope and in a close position on the downhill slope.

The risk of falling might be higher on the downhill slope condition than on the uphill slope condition because the most frequent unexpected postural change has been found to be posterior breakdown caused by the short distance between the WRE user and the 4WW [17]. Paraplegics can maintain a standing posture with a sufficient extension of the hip joint, called the “C-posture”, while wearing a knee–ankle–foot orthosis that is similar in structure to the WPAL. The main characteristic of the C-posture is the center of mass of the body being positioned behind the hip joint. [27]. It is more difficult for patients with SCI to maintain an appropriate C-posture while walking on a downhill slope because of the short distance between the WRE user and the 4WW. Furthermore, backward falls are associated with a higher frequency of sustaining a hip fracture than forward falls [28].

The transition between level and uphill (L-Up and Up-L) had significantly different distances between the WRE user and the 4WW compared to that in the level condition, whereas no significant differences were found between distances during the transition between level and downhill (L-Dn and Dn-L) compared to that in the level condition. This result is a novel finding of the present study and may be related to the walking strategy of WPAL users to avoid moving too close to the 4WW on L-Up and Up-L. Additionally, the forward force of the 4WW on L-Up and Up-L might be greater than the backward force of the 4WW on these. In contrast, the results of the distances between the WRE user and the 4WW on L-Dn and Dn-L could be because of another walking strategy in which the user maintains an appropriate distance by means of an equilibrium between the force caused by the walker descending and that caused by pulling the 4WW.

A feedback system could contribute to achieving safe and independent walking with WRE. For example, warning sounds indicating the detection of the inappropriate distance between the WRE user and the 4WW may function to alert the user to change their posture during walking. In addition, sounds indicating that the distance between the WRE user and the 4WW is within the appropriate range may lead to early proficiency in walking movements with the WRE. The feedback emitted by WRE during walking might be a key complementary motor learning and motor control strategy [29]. A previous study reported that a decrease in the frequency of all unexpected postural changes during gait training may result from an improvement in walker operation, adjustment of the center of gravity, and the ability to predict lower leg position from residual sensory information [17].

This study has three limitations. First, the mechanisms of the present results are unclear. Future studies should include a more detailed analysis using electromyography of the upper limb during 4WW control, three-dimensional motion analysis, and grand reaction force during slope walking. Through this simultaneous measurement, the direction and timing of the force and muscle activation patterns involved in the generation of the force will be clarified. This may be useful for the development of a practical method for walker operation. A previous study reported that slope inclination affected the ground reaction force during the transition between level and slope walking conditions of healthy people without walking aids [30]. Second, the present study examined only one type of 4WW. Other studies have reported the use of various types of walkers such as rolling, pick-up, and platform types to support standing balance during gait with WRE [14]. Different types of walker specifications of different devices may yield different results. In addition, the use of the smart assist walker might be effective in maintaining the distance between the walker and the WRE user [31]. The functionalities of smart walkers include guiding strategies, navigation systems, and fall prevention modules [32]. Future studies using smart walkers will reveal the effects of gravity on 4WWs. Third, the present study did not examine time-course changes in the distance between the WRE user and the 4WW during the process of motor learning because the study only recruited healthy volunteers who had experienced WPAL walking. Thus, the distance between the WRE user and the 4WW during the initial gait training is not clear. A previous study reported that during 10 days of initial gait training, all types of unexpected postural changes showed a gradual decrease in their incidence [17]. It is necessary to investigate the effect of differences in proficiency on the distance between the WRE user and the 4WW, as future robot development requires a function to prevent falling during initial gait training of patients.

## 5. Conclusions

In conclusion, the present study measured and compared the distances between WRE users and their 4WWs during level and slope walking conditions. The distances increased in uphill slope conditions and decreased in downhill slope conditions. In walking with WREs, these changes in the distance between the WRE user and the 4WW might lead to an increase in the risk of falling forward on an uphill slope and backward on a downhill slope compared to a level surface. These findings will be useful for developing fall prevention and feedback systems to facilitate best walking practices using WREs during various walking conditions.

## Data Availability

The data that support the findings of this study are available from the corresponding authors, Tan K. or Koyama S., upon reasonable request.
